# Effects of chronic nicotine administration on behavioral parameters and Na⁺/K⁺-ATPase mRNA expression in the brains of male Swiss Albino mice exposed to chronic stress during adolescence

**DOI:** 10.1186/s40360-025-01004-z

**Published:** 2025-10-27

**Authors:** NourAllah Ahmed, Mohamed Ahmed, Assem Ragab, Mohamed Kamal, Abdullrahman Elsayed, Moustafa Sayed

**Affiliations:** 1 Center of Drug Research and Development (CDRD), Faculty of Pharmacy, The British University in Egypt (BUE), El-Sherouk City, Cairo Egypt; 2 Department of Pharmacology, Faculty of Pharmacy, October 6th University, Giza, Cairo Egypt; 3 Pharmacology and Biochemistry Department, Faculty of Pharmacy, The British University in Egypt (BUE), El-Sherouk City, Cairo Egypt; 4 Clinical Pharmacy Practice Department, Faculty of Pharmacy, The British University in Egypt (BUE), P.O. Box 43, El-Sherouk City, Cairo Egypt; 5 Biochemistry Department, Faculty of Pharmacy, Ain Shams University, El-Abaseya, Cairo Egypt

**Keywords:** Social defeat, Chronic stress, Nicotine, Na+ /K + ATPase

## Abstract

**Background:**

Stress during adolescence leads to the development of major stress-related diseases. The use of psychoactive substances and most psychiatric disorders have been linked to cigarette smoking during adolescence. Na+/K + ATPase isoforms are related to memory formation, stress activity and psychological disorders. Moreover, nicotine downregulates Na+/K + ATPase α2 isoform in rats’ brains. However, its connection to social stress and nicotine addiction is still unclear. Here we aimed for elucidating the effects of nicotine on behavioral parameters and Na+/K + ATPase mRNA in brains of male Swiss Albino mice exposed to chronic stress during adolescence.

**Methods:**

Adolescent male mice were exposed to the sensory contact model (SCM) for 12 days. Nicotine doses (0.1 and 1 mg/kg) were administered intraperitoneally daily for 14 days after SCM. Distance traveled in open field (OF), time spent in open arms in elevated plus maze (EPM), percentage spontaneous alteration in Y-maze and immobility time in forced swimming test (FST) were used to assess locomotor activity, anxiety, spatial memory and depression, respectively. In addition, qRT-PCR was conducted to measure the mRNA levels of Na+ /K + ATPase isoforms in the brain striatum. Group comparisons were analyzed using one-way analysis of variance (ANOVA).

**Results:**

The significantly increased locomotor activity induced by SCM in the aggressive group was significantly reduced by both nicotine treatments. In EPM test, both aggressive and defeated groups showed a significant state of anxiety-like behavior compared to control group which was significantly reduced by 0.1 mg/kg of nicotine. Aggressive and defeated groups showed significant depressive-like response in FST which was almost abolished in defeated group receiving 0.1 mg/kg treatment; however, 1 mg/kg treatment showed the opposite result in the defeated group. SCM significantly increased Na+/K + ATPase-α2 mRNA levels in aggressive and defeated groups compared to control group. In comparison to the control group that received saline, both nicotine treatments significantly increased the levels of Na+/K + ATPase-α2 mRNA in the other two control groups.

**Conclusion:**

This study demonstrates nicotine effects on stress-induced behavioural and molecular alterations in a dose dependent manner. By assessing behavioural outcomes and examining the Na⁺/K⁺ ATPase-α2 expression levels, the study advances understanding of nicotine interactions with chronic stress during adolescence, offering insights into mechanisms underlying nicotine’s behavioral effects.

**Clinical trial number:**

Not applicable.

## Background

Adolescence is a critical developmental stage marked by increased novelty seeking and risk-taking behavior [1]. These behaviors and impulsiveness make adolescents vulnerable to experimentation and substance abuse especially smoking initiation [1, 2]. Tobacco smoking has been closely linked to behavioral alterations, mood disorders and increased risk of addiction and psychiatric disorders such as anxiety and depression [3, 4]. This association between early nicotine dependence in adolescence and mental health deterioration has been largely investigated in various studies [5, 6]. Interestingly, researchers found that individuals with pre-existing mental health conditions were more susceptible to early smoking initiation, developing stronger nicotine dependence with a higher rate of cessation failure [7–9]. Moreover, multiple studies highlighted a multifaced link between smoking and emotional psychopathology, especially depression [10]. This linkage has been investigated through different stages of smoking from initiation [11], progression to regular smoking [12], development and maintenance of nicotine dependence [13], as well as increasing the risks of smoking cessation failure [14, 15].

Studying the effect of stress on nicotine consumption or dependence confirmed that stress contributes significantly to the number of cigarettes consumed and was strongly connected to tobacco dependency and relapse [16–20]. Different animal models, such as social defeat models, were proved valuable in replicating the psychological effects of anxiety and depression in humans [21–24]. Social defeat model involves repeated confrontation between paired rodents, where one consistently exhibits dominant (aggressive) behaviour while the other adopts a submissive (defeated) role [25]. A prolonged exposure to such stress paradigms showed behavioural impairments in the studied animals along with reduced locomotor activity and depressive phenotypes such as social withdrawal and anhedonia [26, 27].

Na+/K + ATPase pump is proven to play an essential role in neuronal signalling, molecular translation and maintaining the electrochemical gradient [28, 29]. There are 2 main components for Na+/K + ATPase pump which are the catalytic α-subunit and the regulatory β- subunit, the α-subunit exists in four different isoforms, α1 through α4, where α2 and α3 isoforms are expressed in the brain tissues [30, 31]. Interestingly, nicotine downregulates α_2_ isoform of Na+/K + ATPase in rats’ brains [32]. Although studies proved Na+/K + ATPase pump connection to memory formation, stress activity and psychological disorders, its relation and or connection to social stress and nicotine addiction together is still not clear [33–36].

Adolescent stress exposure is a profound risk factor for the development of many stress-related illnesses [37]. Cigarette smoking has been connected to the majority of psychiatric problems, and can affect the substantial dynamic morphological and cellular brain changes that occur between adolescence and the early 20s [38]. Memory formation, stress activity, and psychological problems are associated with Na+/K + ATPase isoforms, particularly alpha-2 and alpha-3 [39]. Moroever, nicotine downregulates α_2_ isoform of Na/K ATPase in rat’s blood-brain barrier and brain [32]. It is known that all three Na/K ATPase α-subunit isoforms exist in neurons from the striatum [40]. The phosphorylation state and activity of ion pumps, voltage-gated ion channels, neurotransmitter receptors, and transcription factors are regulated by signaling pathways in the striatum and these pathways are also influenced by centrally acting medications, such as antipsychotics and substances of abuse [41]. Additionally, the striatum is divided into dorsal and ventral divisions. While the ventral region is involved in reward and aversion processes, the dorsal region governs motor and executive activities [42]. Interestingly, Jane Rose et al. has reported that chronic exposure to nicotine is associated with reduced reward-related activity in the striatum but not the midbrain [43]. However, Na+/K + ATPase isoforms expression in the striatum and its connection to social stress and nicotine addiction is still unclear making it a new therapy target for both stress related disorders and smoking cessation once its connection to both is fully revealed and understood.

In this study we aimed for examining the effects of chronic exposure to stress and nicotine on behavioural changes along with the Na+ /K + ATPase alpha-2 and alpha-3 mRNA expression levels in the striatum of male mice.

## Methods

### Animals

Experimental procedures were carried out on ninety weaned male Swiss albino mice (postnatal day 22, PND 22), provided by the director of the animal house facility at the faculty of Pharmacy in the British University in Egypt. The animals were housed in groups of 4 to 6 per cage under standard laboratory conditions. Mice were then given an adaptation period of 7 days (PND22-PND29) without any experimental intervention. After the adaptation period the experimented mice assigned to the sensory contact model groups were then housed individually for 5 days to eliminate any social effects (PND29-PND34). Tall housing and handling procedures were designed to minimize animal suffering and were conducted in accordance with the guidelines for animal experiments at the Faculty of Pharmacy, the British University in Egypt and approved by the institution’s Ethical Committee.

### Sensory contact model (SCM)

The model was adapted from Kudryavtseva et al. [44] and further refined from our previous paper Ibrahim et al. [45], where weight-matched pairs of adolescent male mice were housed in stainless steel cages (28 × 14 × 10 cm). The cages were separated by a transparent and perforated Plexiglas partition that allows visual and olfactory yet preventing physical contact. The paired mice remained in this setting for 48 h to be familiarized with the sensory contact setting. On PND36, the divider was removed once daily for 10 min at the same timing (10:00–11:00 am) for 12 consecutive days (PND36-48). During the examination period, one male was seen to dominate over his pair exhibiting aggressive behaviors (e.g. chasing, biting, etc.). While the other male showed submissive behavior such as upright postures, withdrawal, sideways, freezing or lying on the back. The confrontations between the experimented mice were discontinued if the aggression lasted beyond 3 min. After every session, the winner remained resident in their original compartments while the defeated/submissive mice were re-paired with another dominant mouse in an unfamiliar cage.

### Experimental design

Following the social confrontation model (SCM), adolescent mice were categorized into one of three experimental conditions: aggressive, defeated, or non-stressed controls. Mice were classified as aggressive or defeated based on the behaviour displayed during the confrontation sessions. For each experimental condition, mice were randomly assigned to one of three treatment groups receiving either saline (vehicle), nicotine at a dose of 0.1 mg/Kg or nicotine at a dose of 1 mg/Kg. All treatments were administered intraperitoneally (i.p.) once daily for 14 consecutive days from PND 48 to PND 61. The first injection administered two hours after the final SCM session.


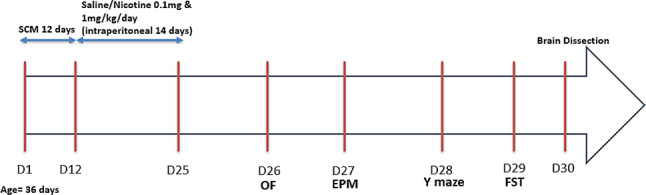


### Drugs

The nicotine administered in this experiment was (-)-Nicotine hydrogen tartrate salt (Sigma-Aldrich, St. Louis, MO), freshly dissolved in physiological saline (Patterson Veterinary, Devens, MA) prior to each injection. Nicotine dosages were calculated based on the nicotine free base and expressed in mg/kg of body weight. All intraperitoneal (i.p.) injections were administered at a fixed volume of 10 ml/kg body weight, as recommended by Turner et al. [46]. The injection volume was adjusted based on each mouse’s weight to ensure precise dosing. Nicotine solutions were freshly prepared at concentrations of 0.01 mg/mL for the 0.1 mg/kg dose and 0.1 mg/mL for the 1.0 mg/kg dose, based on the nicotine free base equivalent. As the compound used was (–)-nicotine hydrogen tartrate salt (MW = 498.44 g/mol), we applied a correction factor of ~ 3.1 to account for the molecular weight difference from nicotine free base (MW = 162.23 g/mol), following Matta et al. recommendations [47]. This ensures that the reported doses reflect the actual amount of nicotine free base received by each mouse. Nicotine was injected intraperitoneally daily at 9:00 a.m. for 14 consecutive days (PND48–PND61) following the final social confrontation session.

### Nicotine doses selection

With respect to absolute nicotine effects, it is reported that lower doses (0.1 mg/kg) may induce locomotor stimulant effects, while hihger doses (1 mg/kg) may lead to long-lasting locomotor suppression [48]. Moreover, lower doses (0.1 mg/kg) have been shown to have anxiogenic like effects initially, but tolerance can develop, leading to an anxiolytic like effects with chronic exposure [49]. Also low doses of nicotine (0.1 mg/kg) treatment can sometimes buffer the negative behavioral consequences of stress [50]. Finally, social defeat stress, has been reported to have an impact on nicotine-induced locomotor responses, with some studies suggesting that episodic and continuous social defeat stress have contradicting outcomes on nicotine’s locomotor stimulant effects [51]. Thus, it is imporant to reveal the effects of both low and high doses of nicotine only after stress exposure.

### Behavioral assessment

#### Open-field test (OF)

The open field test was used to assess the locomotor activity, and the apparatus used was a black Plexiglas chamber measuring (40 × 40 × 30 cm). The test was performed under dim light conditions (10 lx). Each mouse was placed individually in the center of the arena and was recorded for 5 min while exploring the arena. The arena was cleaned thoroughly after each session with 70% propanol as described by Weiss et al. [52]. The behavioral parameters recorded were analyzed using Any Maze^®^ video tracking software (Stoelting co., USA) and the following parameters were investigated time spent in the center, total immobility time and total distance travelled.

### Y-maze test

The Y-maze apparatus was the apparatus of choice to test the spatial working memory. The apparatus consisted of three identical black arms with the following dimensions (l × w × h = 35 × 6 × 15 cm) and equal angles (120°) named A, B, and C to form the Y-shape. At the beginning of each test, the mouse was placed at the entrance of one arm of the apparatus and given a duration of 10 min to explore the maze freely. Spontaneous alternation was defined as consecutive entries into three different arms (an arm choice differing from the previous two). The entry of an arm was considered valid when the forelimbs and hindlimbs of the mouse fully entered the arm. The maze was cleaned with 70% ethanol between trials to minimize olfactory cues. The spontaneous alternation percentage (SAP) was calculated using the following equation: SAP = [(number of alternations)/ (total arm entries − 2)] × 100 [53].

### Elevated plus maze test (EPM)

Anxiety-like behavior was tested using the elevated plus maze test, following the design described by Bourin et al. [54]. The maze structure consists of a central platform (5 × 5 cm) elevated 40 cm above the floor, with two opposing open arms with the following dimensions (5 × 30 cm) and two enclosed arms of the same length and width but with high walls of 15 cm. The mouse examined is placed on the central platform and allowed to explore the maze for 5 min. The animal behavior was then recorded and analyzed considering the time spent in the open arms versus the closed ones to indicate anxiety-like responses from the tested mice. The maze was cleaned with 70% ethanol between trials to minimize olfactory cues.

### Forced swimming test (FST)

The forced swimming test was used to assess the behavioral despair shown by the experimented mice following the methodology described by Yin et al. [55]. The experimented mice were placed individually in the apparatus which was a glass cylinder (20 cm diameter, 50 cm height) filled with tap water at 25 ± 1 °C with 30 cm depth to eliminate tail support. The test was recorded and lasted for 6 min with the first 2 min serving as a pre-test to allow the mouse to adapt to the new surroundings and then the immobility time was counted during the last 4 min. At the end of the test, mice were dried gently using paper towels, placed under heat lamps to restore their body temperature and then returned to their home cages. After every session, the water used was changed to avoid any influence and maintain consistency across the trials.

### Quantitative real time PCR (qRT-PCR)

#### Tissue extraction

Brain tissues were extracted one day after the final behavior assessment. Mice were euthanized by decapitation in reference to the following literature [56], and the striatum was isolated via microdissection following the exact procedure described by Chiu et al. [57]. The collected tissues were snap-frozen in liquid nitrogen and stored at -80 °C, and the total RNA was extracted using Trizol Reagent (Invitrogen, USA) as per the instructions provided by the manufacturer. UV–Vis Spectrophotometer Quawell q5000 (Quawell, USA) was used to quantify the isolated RNA spectrophotometrically, with acceptable purity defined by a 260/280 absorbance ratio between 1.8 and 2.0. Genomic DNA was removed by treating the RNA samples with RNase-free DNase (Thermo Scientific, USA) to remove any contamination. RevertAid First Strand cDNA Synthesis Kit (Thermo Scientific, USA) was used to synthesize complementary DNA (cDNA) from the DNase-treated RNA. Gene expression was analyzed using quantitative real-time PCR (qRT-PCR) using gene-specific primers (listed in Table 1) and Maxima SYBR Green qPCR Master Mix (2X) (Thermo Scientific, USA). All primers were designed by the authors using Primer3 online free primer design tool (https://primer3.ut.ee/) and using the sequence of gene adopted from Ensembl Genome browser (https://www.ensembl.org/index.html) following all criteria of good primers proposed by the software. Afterwards, each pair of primers was checked on UCSC genome browser In-Silico PCR tool (https://genome.ucsc.edu/cgi-bin/hgPcr) to pick the genomic DNA which as further applied to Basic Local Alignment Search Tool (BLAST) tool of NCBI (https://blast.ncbi.nlm.nih.gov/Blast.cgi). Final primer pair which fulfils good primer criteria and picks only one hit on BLAST was used in this study for the corresponding genes. All qRT-PCR experiments were conducted using StepOnePlus Real-Time PCR System (Applied Biosystems, USA). Relative RNA expression levels in relation to β-actin as the house keeping gene were calculated using the threshold cycle (Ct) values detected by the instrument using the formula 2 − ΔCt. The cycling protocol was as follows: initial denaturation at 95 °C for 10 min, followed by 40 amplification cycles of denaturation at 95 °C for 15 s and annealing/extension at 60 °C for 1 min.

**Table 1:** Primers sequences used in qRT-PCR experiment

Gene	Forward primer	Reverse Primer
ATP1α2	5′-GTC CCT GAG GAT CTC ATC CA-3′	5′-TGT GGG CAT CAT ATC AGA GG-3′
ATP1α3	5′-GAT GAT ACC CAC ACC CTT GG-3′	5′-TCA CCA CAG ACA ACC TTT GC-3′
β-actin	5′-CTT GCT CTG GGC CTC GTC − 3′	5′-GGC TGT ATT CCC CTC CAT C-3′

### Statistical analysis

For the behavioral assays, open field, Y-maze, EPM and FST, the scorer of the data was blind to treatment groups. The data generated from both behavioral tests and qRT-PCR tests were expressed as mean ± standard error of mean (SEM). Group comparisons were analyzed using one-way analysis of variance (ANOVA), followed by Tukey’s post-hoc test for multiple comparisons. Prior to ANOVA, variance equality was verified with the Brown-Forsythe test. A probability level (p-value) of less than 0.05 was considered statistically significant. All statistical procedures and calculations were conducted using IBM SPSS version 17 (IBM Corp., USA), and graphical representations were generated using GraphPad Prism version 5 (GraphPad Software Inc., USA).

## Results

### Open field test (OF)

**Fig. 1:**
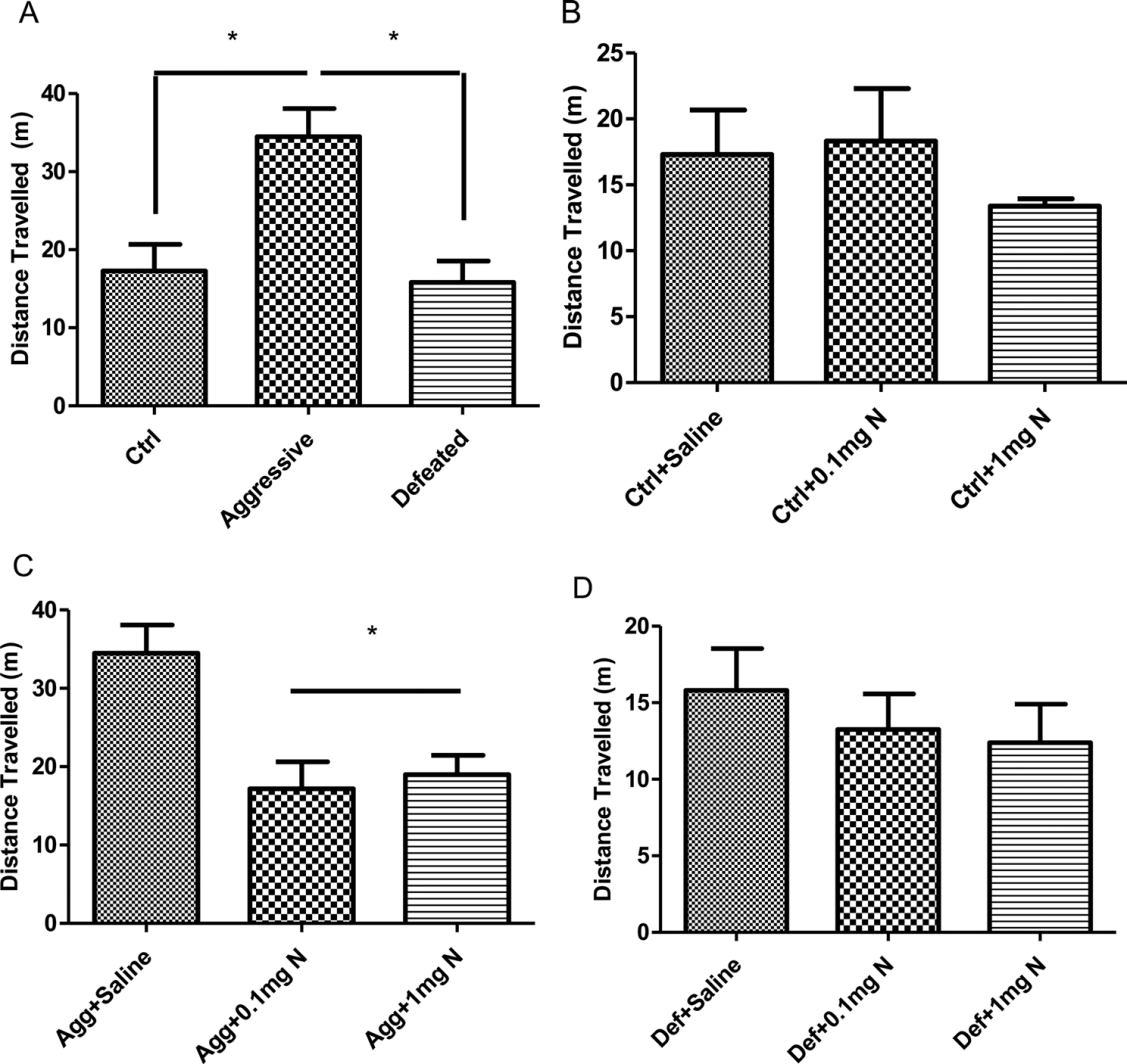
Effect of 14 days’ nicotine administration (0.1 and 1 mg/kg/day i.p.) on the total distance travelled by mice for 5 min observation session (*n* = 6–8). (**A**) Distance travelled by control, aggressive and defeated groups of the SCM. (**B**) Distance travelled by control groups receiving either saline, 0.1mgN or 1mgN. (**C**) Distance travelled by aggressive groups of the SCM receiving either saline, 0.1mgN or 1mgN. (**D**) Distance travelled by the defeated groups of the SCM receiving either saline, 0.1mgN or 1mgN. Ctrl: Control group; Agg: Aggressive group; Def: Defeated group; 0.1mgN: 0.1 mg nicotine; 1mgN: 1 mg nicotine. *: means are significantly different at *p* < 0.05

The results obtained from the OF test showed that SCM significantly increased the distance travelled by the aggressive group when compared to the control and defeated group, indicating significant increase in the locomotor activity (Fig. 1A), and higher tendency of aggressive mice to move and explore in a novel environment. However, a significant reduction in the distance travelled was observed in the aggressive groups receiving nicotine treatment (0.1 and 1 mg/kg) when compared to the aggressive group that received saline only (Fig. 1C). Further, there was no significant difference observed in the locomotor activity among the control groups receiving saline or nicotine (Fig. 1B). Similarly, no significant alterations were noticed in the defeated groups (Fig. 1D).

### Y maze

**Fig. 2:**
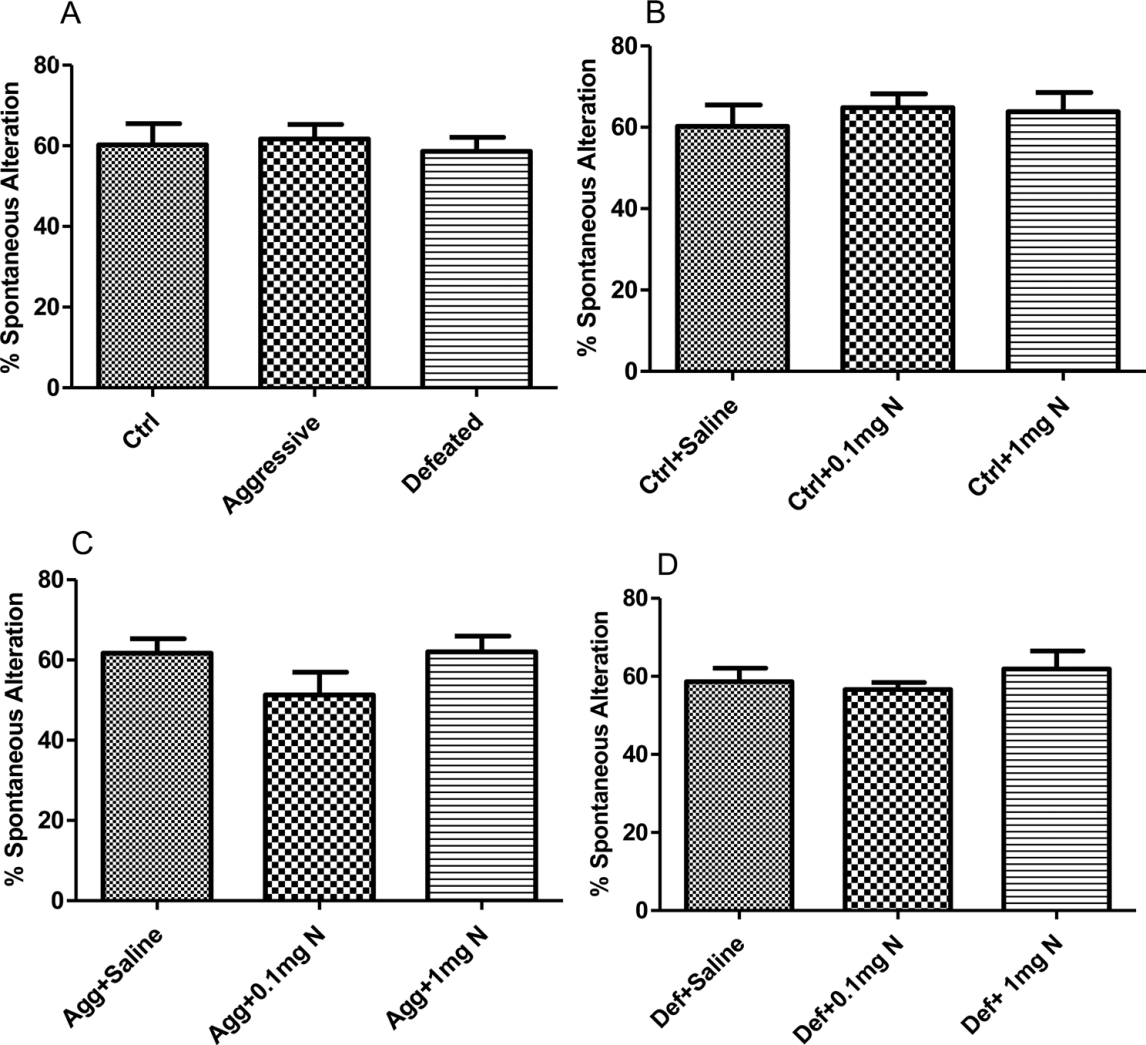
Effect of 14 days’ nicotine administration (0.1 and 1 mg/kg/day i.p.) on the % spontaneous alteration in the Y-maze test (*n* = 6–8). (**A**) % spontaneous alteration in Y maze by control, aggressive and defeated groups of the SCM. (**B**) % spontaneous alteration in Y maze by control groups receiving either saline, 0.1mgN or 1mgN. (**C**) % spontaneous alteration in Y maze by aggressive groups of the SCM receiving either saline, 0.1mgN or 1mgN. (**D**) % spontaneous alteration in Y maze by the defeated groups of the SCM receiving either saline, 0.1mgN or 1mgN. Ctrl: Control group; Agg: Aggressive group; Def: Defeated group; 0.1mgN: 0.1 mg nicotine; 1mgN: 1 mg nicotine. Data is presented as the percentage of spontaneous alterations

The control group exhibited almost no difference in the percentage of spontaneous alternation when compared to aggressive and defeated groups (Fig. 2A). Additionally, nicotine administration at both doses (0.1 mg/kg and 1 mg/kg) did not alter spontaneous alternation behavior in control mice (Fig. 2B), aggressive mice (Fig. 2C), or defeated mice (Fig. 2D). Which indicates that neither chronic stress nor nicotine treatment influences spatial memory.

### Elevated plus maze (EPM)

**Fig. 3:**
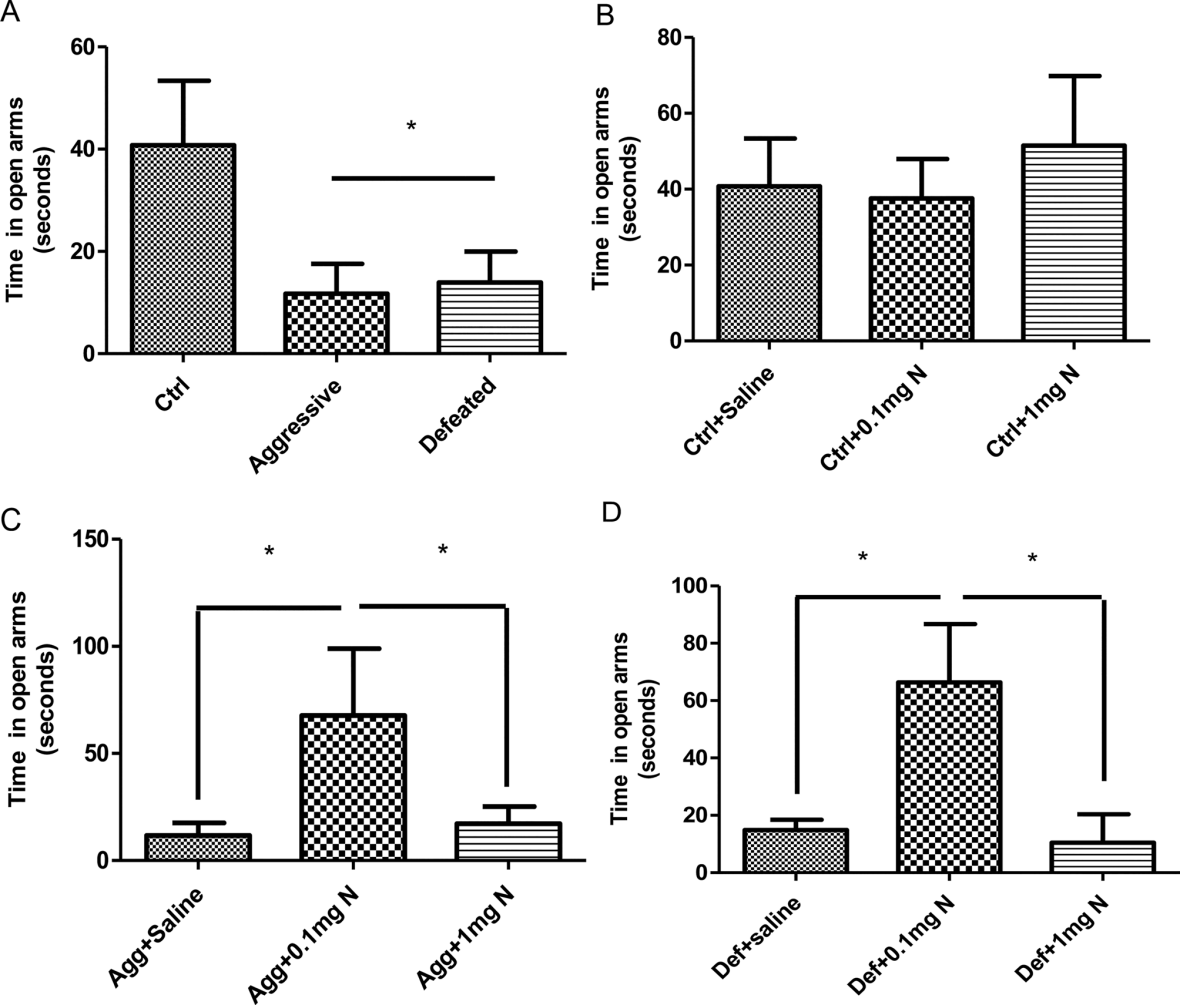
Effect of 14 days’ nicotine administration (0.1 and 1 mg/kg/day i.p.) on the time spent in open arms in elevated plus-maze test (EPM) (*n* = 6–8). (**A**) Time spent in the open arm by control, aggressive and defeated groups of the SCM. (**B**) Time spent in the open arm by control groups receiving either saline, 0.1mgN or 1mgN. (**C**) Time spent in the open arm by aggressive groups of the SCM receiving either saline, 0.1mgN or 1mgN. (**D**) Time spent in the open arm by the defeated groups of the SCM receiving either saline, 0.1mgN or 1mgN. Ctrl: Control group; Agg: Aggressive group; Def: Defeated group; 0.1mgN: 0.1 mg nicotine; 1mgN: 1 mg nicotine. *: means are significantly different at *p* < 0.05

In the EPM, SCM caused a significant decrease in the time spent (*p* < 0.05) in the open arms for both aggressive and defeated groups than the control group (Fig. 3A), revealing a higher state of anxiety. Remarkably, an anxiolytic-like effect was shown in the control group receiving 1 mg/kg of nicotine treatment, expressed by an increase in the time spent in open arms when compared to the control group receiving saline, however it was not significant (Fig. 3B). Notably, a significant anxiolytic-like effect was demonstrated in both aggressive and defeated groups receiving 0.1 mg/kg of nicotine, which is again manifested by significant increase in the time spent in the open arms when compared to the other groups (Fig. 3C, D).

### Forced swimming test (FST)

**Fig. 4:**
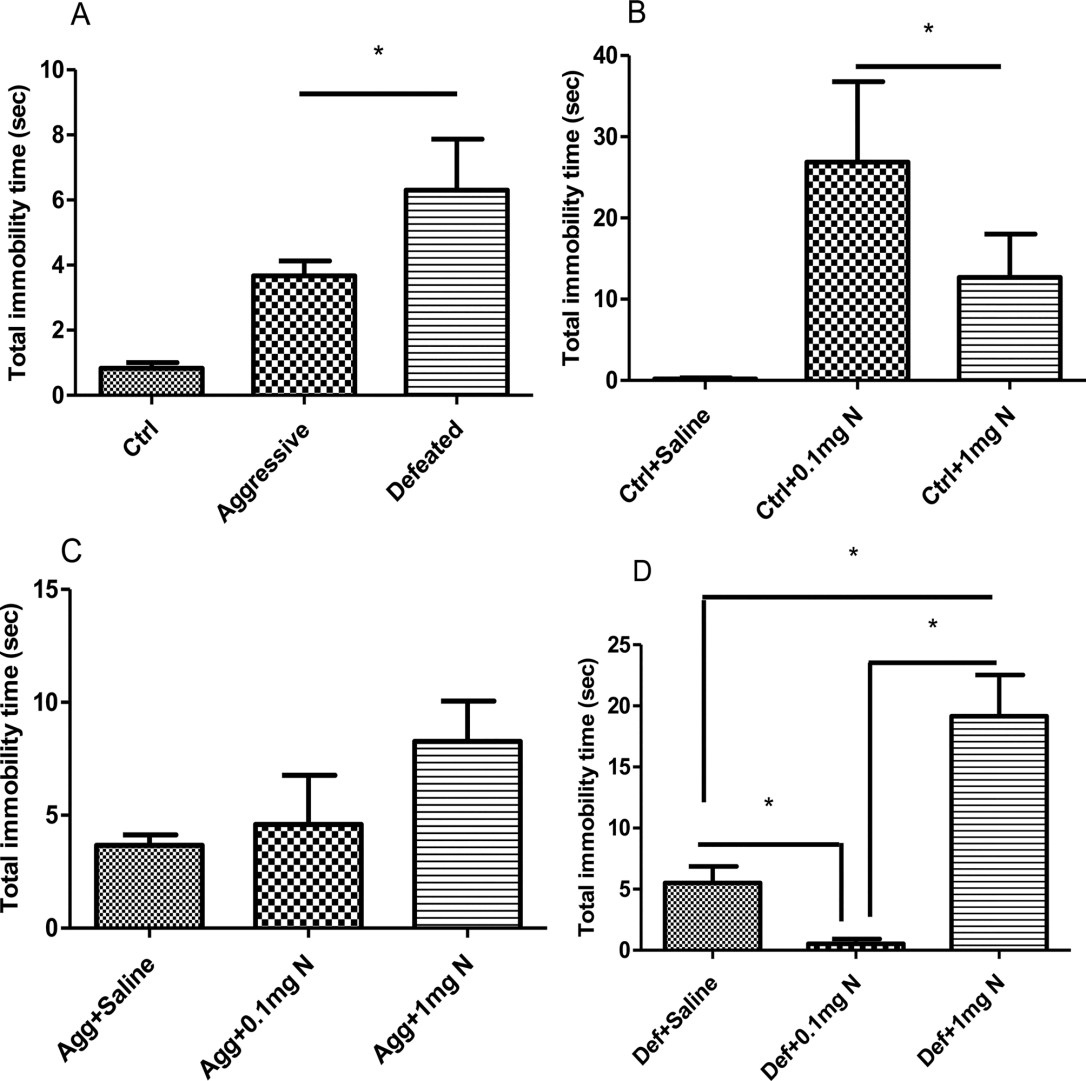
Effect of 14 days’ nicotine administration (0.1 and 1 mg/kg/day i.p.) on the total immobility time in the forced swimming test (*n* = 6–8). (**A**) Total immobility time spent by control, aggressive and defeated groups of the SCM. (**B**) Total immobility time spent by control groups receiving either saline, 0.1mgN or 1mgN. (**C**) Total immobility time spent by aggressive groups of the SCM receiving either saline, 0.1mgN or 1mgN. (**D**) Total immobility time spent by the defeated groups of the SCM receiving either saline, 0.1mgN or 1mgN. Ctrl: Control group; Agg: Aggressive group; Def: Defeated group; 0.1mgN: 0.1 mg nicotine; 1mgN: 1 mg nicotine. *: means are significantly different at *p* < 0.05

For FST, the results were recorded to present the mean total of the immobility time for the control and treated groups (Fig. 4). A significant increase in the total immobility time was observed in both the aggressive and defeated when compared to the control group (Fig. 4A). Further, the total immobility time in the control groups receiving both doses of nicotine (0.1 mg/kg and 1 mg/kg) significantly increased, highlighting that nicotine treatment had a depressive-like effect on normal mice when compared to the control group (Fig. 4B). On the other hand, no significant alterations were observed on the depressive-like response or in the total immobility time in the aggressive group receiving different doses of nicotine (Fig. 4C). Interestingly, the depressive response in the defeated group was abolished when they received a nicotine dose of 0.1 mg/kg. Oppositely, the higher dose of nicotine (1 mg/kg) in the defeated groups had depressive effects expressed by the significant increase in the total immobility time (Fig. 4D).

### Na⁺/K⁺ ATPase mRNA expression level in striatum

**Fig. 5:**
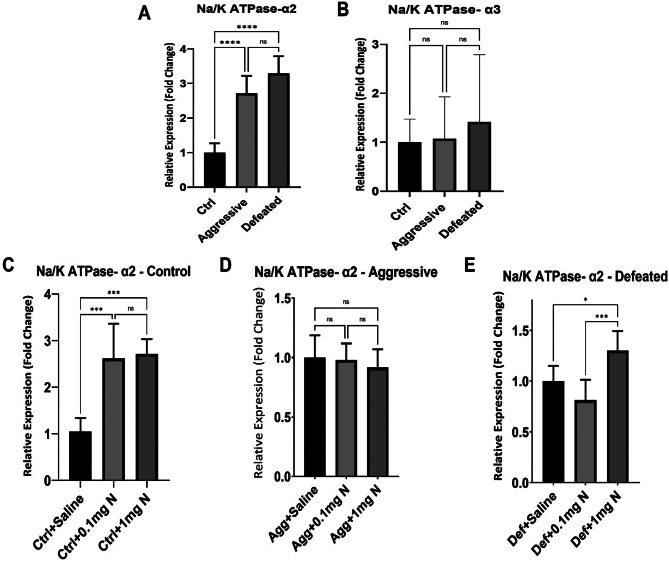
Gene expression of Na⁺/K⁺ ATPase-α2 and Na⁺/K⁺ ATPase-α3 in different groups (*n* = 5). (**A**) Relative expression levels of Na⁺/K⁺ ATPase-α2 in control, aggressive and defeated groups of the SCM. (**B**) Relative expression levels of Na⁺/K⁺ ATPase-α3 in control, aggressive and defeated groups of the SCM. (**C**) Relative expression levels of Na⁺/K⁺ ATPase-α2 in control group receiving either saline, 0.1mgN or 1mgN. (**D**) Relative expression levels of Na⁺/K⁺ ATPase-α2 in aggressive group of the SCM receiving either saline, 0.1mgN or 1mgN. (**E**) Relative expression levels of Na⁺/K⁺ ATPase-α2 in defeated group of the SCM receiving either saline, 0.1mgN or 1mgN. Ctrl: Control group; Agg: Aggressive group; Def: Defeated group; 0.1mgN: 0.1 mg nicotine; 1mgN: 1 mg nicotine. *: means are significantly different at *p* < 0.05. ***: means are significant different at *p* < 0.001. ****: means are significantly different at *p* < 0.0001

**Fig. 6:**
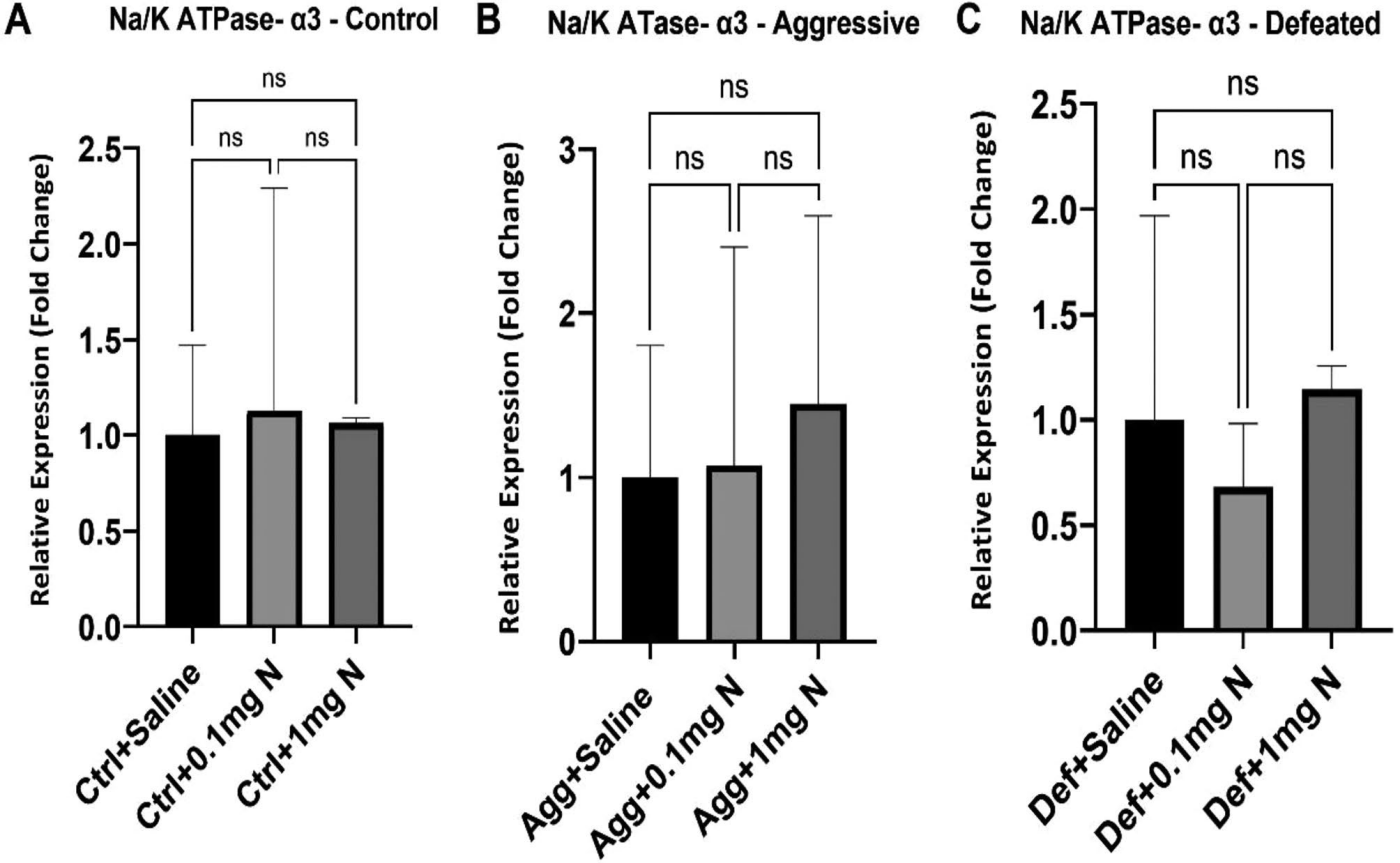
Gene expression of Na⁺/K⁺ ATPase-a3 in different groups (*n* = 5). (**A**) Relative expression levels of Na⁺/K⁺ ATPase-α3 in control group receiving either saline, 0.1mgN or 1mgN. (**B**) Relative expression levels of Na⁺/K⁺ ATPase-α3 in aggressive group of the SCM receiving either saline, 0.1mgN or 1mgN. (**C**) Relative expression levels of Na⁺/K⁺ ATPase-α3 in defeated group of the SCM receiving either saline, 0.1mgN or 1mgN. Ctrl: Control group; Agg: Aggressive group; Def: Defeated group; 0.1mgN: 0.1 mg nicotine; 1mgN: 1 mg nicotine

Figure 5 shows the effect of SCM on the expression of Na⁺/K⁺ ATPase-α2, for example Na⁺/K⁺ ATPase-α2 was significantly elevated (3 folds) on both aggressive and defeated groups when compared to the control group (Fig. 5A). Yet, no significant difference was observed between the aggressive and the defeated groups (control: 1.00 ± 0.12; Aggressive: 2.71 ± 0.21; defeated: 3.29 ± 0.19). On the other hand, when studying the expression of Na⁺/K⁺ ATPase-α3, no significant difference was observed in all the study groups (control: 1.00 ± 0.11; aggressive 1.07 ± 0.20; defeated:1.415 ± 0.32) (Fig. 5B). The effect of nicotine treatment (0.1 mg/kg and 1 mg/kg) on the expression of Na⁺/K⁺ ATPase-α2 was then studied in the control, aggressive and defeated groups. Na⁺/K⁺ ATPase-α2 expression with both doses of nicotine (0.1 mg/kg and 1 mg/kg) was significantly elevated to up to three folds in the control mice in comparison to the control group receiving saline (control with saline: 1.05 ± 0.12; control with 0.1 mg/kg nicotine: 2.62 ± 0.32; control with 1 mg/kg nicotine: 2.71 ± 0.13) (Fig. 5C). In the aggressive group, neither the mice receiving 0.1 mg/kg or 1 mg/kg treatments showed any significant change in Na⁺/K⁺ ATPase- α2 expression when compared to the ones receiving saline (Agg + saline: 1.00 + 0.08; Agg + 0.1 mg/kg: 0.97 ± 0.06; Agg + 1 mg/kg:0.92 ± 0.06) (Fig. 5D). Remarkably, Na⁺/K⁺ ATPase-α2 expression was slightly (30%) yet significantly elevated in the defeated group receiving 1 mg/kg nicotine when compared to the defeated groups receiving saline and 0.1 mg/kg nicotine (Def + saline: 1.00 ± 0.06; Def + 0.1 mg: 0.81 ± 0.08; Def + 1mgN: 1.31 ± 0.07) (Fig. 5E). Alternatively, the expression of Na⁺/K⁺ ATPase-α3 failed to show any significant change upon administration of either doses of nicotine in negative control, aggressive or even defeated groups when compared to their corresponding saline administered groups (Fig. 6).

## Discussion

Since the identification of stress in the 1930s by Hans Seyle, chronic stress has been associated with different somatic and psychiatric disorders [58]. This enticed the development of different animal models to study the impact of stress on behavior; out of which social defeat stress (SDS) has gained recognition in its ability to induce depression and anxiety-like phenotypes in the studied animals [59, 60]. Despite the numerous studies that have been conducted to test anxiety-or depression-related reactions following stress exposure, the results remain inconsistent. Some studies have reported an increase in anxiety and depression-like phenotypes after stress exposure, while others reported either a decreased response or no significant effects [61–63]. These contradictions in the findings highlights the influence of experimental variables from the kind of stressor used, the duration and the intensity of the stimulus used to the developmental stage of the animals or the inter-individual variances in stress susceptibility.

Nicotine, the psychoactive substance and leading cause of preventable death, is widely recognized for causing the addictive properties of tobacco [64]. In addition, tobacco dependent users were found to struggle with cognition impairments and changes in their emotional status [65]. Not many studies have examined the direct effects of nicotine as a stressor [66]. One of these studies, investigated the interactions between nicotine and Chronic Unpredictable Mild Stress (CUMS) and reported that CUMS caused aggravation to the neuronal and behavioral effects of nicotine [67]. On the other hand, others have reported antagonistic effects caused by stress with nicotine administration [68–70]. Thus, the relationship between nicotine and different stressors continues to be ambigious. We think that this is the first study to characterize the effects of chronic nicotine administration at two different doses (0.1 and 1.0 mg/kg) on behavioral parameters and Na+/K + ATPase mRNA expression levels in brains of male Swiss Albino mice exposed to chronic social defeat stress during adolescence.

### Interactions of social defeat stress and nicotine on psychomotor activity

Previous studies have reported that social defeat stress leads to reduced locomotor activity, but it is worth mentioning that these studies investigated locomotor activity within 24 h-post-stress, but long-term challenges as in 10 days or more after repeated stress were not clearly explored [51, 71]. In this study, we found that chronic social defeat stress increased the locomotor activity in the aggressive group only. Regarding nicotine-induced locomotor effects, chronic administration of 0.1 and 1.0 mg/kg of nicotine was found to promote locomotor suppression in aggressive groups, while no significant psychomotor effects were shown in the other groups (control and defeated groups). Thus, our data suggests that chronic exposure to different nicotine doses may induce conditioned suppression of locomotor activity in aggressive mice only, when they are challenged in the same testing environment. Additionally, our results propose that the outcome of stress exposure on nicotine locomotor effects is different for social defeat stress if it is compared to other types of stressors. For example, when Cruz et al. investigated the effect of chronic stress on nicotine induced locomotor activity in rats, the stimulant effect of nicotine was found to be enhanced by chronic stress [72]. Another study reported an increase in nicotine-induced locomotor activity after the exposure to chronic variable stress [73]. However, Zago et al. reported no significant impact on the locomotor activity of rats, although a similar stress procedure to Cruz et al. was used, including even similar age groups (adolescent and adult) for the studied rats [74].

When comparing the results obtained from the mentioned studies to our results, the variance in sensitivity to repeated stress and/or to nicotine itself, could be attributed to the use of different species (rats versus mice). Moreover, the drug effects seen due to the impact of social defeat stress might depend on the drug tested itself. For instance, in a study correlating the effect of stress on alcohol intake, locomotor sensitization to ethanol was studied, and it was found that repeated episodic defeats did not affect locomotor response to alcohol [75]. While others found that continuous defeat stress in mice caused failure in locomotor stimulation after an ethanol challenge [76]. In addition, when researchers studied the effect of social stress on cocaine intake in mice, they concluded that socially defeated mice exhibited increased motor response to amphetamine and increased affinity towards self-administered cocaine [77].

Our data shows that nicotine treatments (both 0.1 mg/kg and 1 mg/kg) did not alter the brain striatum Na+/K + ATPase α-2 mRNA expression levels in aggressive mice. Multiple models of aggression across species imply that the nicotinic acetylcholine receptor (nAChR) agonist nicotine possesses serenic characteristics and hypolocomotion effects [78]. Our findings could role out the involvement of Na+/K + ATPase in the hypolocomotion effects of nicotine in aggressive mice and support other theories of the nicotinic acetylcholine receptor (nAChR) mediated role in the serenic and hypolocomotion effects of nicotine [79–81]. 

Thus, different consequences of social defeat may be observed according to the defeat protocol and to the drug tested [51]. Different nicotine doses and the timing of stress exposure are two main condition that remain to be studied in the future to elaborate on how social defeat stress may interfere with nicotine effects [82].

### Interactions of social defeat stress and nicotine on spatial memory

Nicotine and social defeat stress (SDS) can have varying effects on spatial memory in mice, with some studies showing impairments and others showing enhancements [83–85]. However, our results showed that neither nicotine nor SDS influenced the spatial memory represented by the percentage of spontaneous alteration in the Y-maze test. Our finding supports others’ work where it has been reported that chronic nicotine treatment in normal rats had no effect on learning and memory [70]. Moreover, Monleón S et al. showed that the highest degree of CSDS in male CD1 mice had no significant differences between groups in the water maze test [83]. In addition, male rats subjected to social defeat stress in adolescence exhibited resilience against the development of stress-induced alterations in spatial memory [86].

### Interactions of social defeat stress and nicotine on anxiety and depression

Stress-anxiety relationship has been heavily studeied [87, 88]. Assessment of anxiety in EPM relies on the time spent percentage in the open arms after stress exposure with a decreased percentage an indicator of an anxiety-like behavior [89]. This was displayed in our findings, which confirmed that chronic social defeat stress significantly reduced the percentage of time spent in the open arms for both defeated and aggressive mice.

Nicotine was found in several studies to influence emotional behavior in different species, especially anxious and depressive like behaviors [90]. For example, nicotine was found to exert anxiolytic and antidepressant like effects whether as a standalone drug or when combined with other drugs [90–96]. Also, nicotine blocked stress-induced impairment in the spatial memory [70]. On the contrary, several studies reported that nicotine administration caused a significant increase in the levels of anxiety and depression [97–100]. It is worth mentioning that a few reports discussed the effect of nicotine on stress-enhanced anxiety [101]. Our findings showed that chronic administration of nicotine at the dose of 0.1 mg/kg decreased the anxiety-like effects in both aggressive and defeated mice. Anxiolytic and anxiogenic like effects of nicotine are believed to be mediated throguh the stimulation or inhibition of β2 nicotinic acetylcholine receptors (nAChRs) with low doses of nicotine to reduce anxiety-like behaviors in mice while higher doses of nicotine can induce anxiety-like behaviors [102]. 

FST was employed to assess the depressive-like effects in the experimented rodents. Prior studies highlighted that nicotine had an antidepressive action represented by decreasing the immobilization time in FST [103]. In our study, nicotine was found to induce depressive like effect manifested by the increased immobility time in all groups at both doses (0.1 and 1 mg/kg), the only exception was observed in the defeated group that received nicotine treatment of 0.1 mg/kg. Yet, the underlying mechanisms behind this observation remain unclear and need further investigations. The previous results highlighted a crucial limiting factor, when it comes to studying the pattern of behavioral changes in rodents, that relies on the type of the applied stress stimulus.

Our data shows that SCM induced depression like effects in both aggressive and defeated groups compared to the control group, which is the exact same outcome seen in the Na+/K + ATPase α2 mRNA expression. Both nicotine doses did show depressive like effects on control groups when compared to the control group receving saline. In addition, the mRNA expression levels of Na+/K + ATPase α2 isoform mRNA increased at both nicotine doses. Nicotine treatment in the aggressive group did not alter the depression like effect when compared to the aggressive group receiving saline which aligns with the Na+/K + ATPase α2 mRNA expression level that did not change. In the defeated groups, nicotine treatment of 0.1 mg/kg showed antidepressant like effects while the high dose showed an opposite effect. Same outcome has been revealed with Na+/K + ATPase α2 mRNA expression levels with the nictone low dose decreasing the Na+/K + ATPase α2 mRNA expression while the high dose increasing the Na+/K + ATPase α2 mRNA expression level. This could propose a link between the depressive and the antidepressive like effects of nicotine through Na+/K + ATPase α2 isoform. Nonetheless, this needs further investigations.

Understanding nicotine’s behavioral effects as a psychoactive component of cigarettes will help explain the continuity of tobacco use to diminish anxiety and stress in humans [104], especially after the astonishing findings of the effect of chronic nicotine administration on locomotor activity and plasma amino acid concentration after stress exposure [105, 106]. Not to mention the numerous human studies that showed increasing smokers’ smoking rate after stress exposure which is paradoxically believed by some to subjectively reduce the stress-related feelings of tension [107, 108]. Regular smokers’ mood swings and anxiety is due to their nicotine dependence which leads to increased feelings of stress comparable to nonsmokers, suggesting that acute nicotine withdrawal increases stress, and nicotine reinstatement relieves the feelings of stress [109].

### Interactions of social defeat stress and nicotine on Na⁺/K⁺ ATPase mRNA expression levels

In reflection to the presented results, we assume that chronic stress increased anxiety and depression levels (shown in EPM and FST) in both aggressive and defeated mice could be attributed to the increased striatal mRNA expression levels of Na+/K + ATPase α2 isoform supported by qRT- PCR (Fig. 5A). It is worthnoting that chronic nicotine treatment of 0.1 mg/kg in defeated mice showed a reduced Na+/K + ATPase α2 isoform expression level (Fig. 5E) in addition to its anxiolytic and the antidepressant like effects discussed previously. Moreover, low and high doses of nicotine treatment in the control group and the high dose nicotine treatment in the defeated group showed an increased striatal Na+/K + ATPase α2 isoform expression level (Fig. 5C and E) in addition to their previously discussed depression induced like effects in both control and defeated mice groups.

Our results, align with previous studies highlighting that increased Na+/K + ATPase α2 isoform expression caused neuroinflammation, degeneration, association with stress induced depression and bi-polar disorder [110, 111]. Moreover, it confirms that genetic alteration in Atp1a2, the encoding gene of Na+/K + ATPase α2 isoform, affects locomotor activity and cause anxiety and depressive-like behavior [112–115].

### Limitations

Despite the strengths of this study in combining behavioral and molecular analyses, several limitations must be acknowledged. First, although the sensory contact model (SCM) is a widely accepted model for inducing social defeat stress, intrinsic variability in aggressor-resident interactions may result in differences in intensity of stress between individual mice. Although this is a reflection of social variety in the real world, it may have impacted the individual differences in behavioral and gene expression results. However, the credibility of our findings is reinforced by proper statistical controls and consistent group-level trends.

Second, the study focused exclusively on male Swiss albino mice, limiting the generalizability of findings across sexes and strains. Given that sex differences significantly influence stress reactivity and nicotine pharmacodynamics, female mice should be included in future research to increase translational relevance.

Third, the use of only two nicotine doses (0.1 and 1 mg/kg) limits the resolution of dose–response relationships. Intermediate or higher doses might have produced additional behavioral or molecular effects that were not captured in this study. Likewise, the route of nicotine administration (intraperitoneal), while allowing precise dosing, does not fully mimic the pharmacokinetics of nicotine exposure via smoking or vaping in humans, which may influence both the intensity and duration of nicotine’s effects.

Finally, while qRT-PCR analysis of Na⁺/K⁺ ATPase isoforms in the striatum provided valuable insight into molecular alterations, other brain areas linked to stress and addiction (e.g., hippocampus, prefrontal cortex, amygdala) were not assessed. Including these brain regions would provide a more comprehensive picture of the neurobiological mechanisms involved.

Future studies should address these limitations by including additional nicotine doses, longer follow-up periods, multiple brain regions, and both sexes, to strengthen our understanding of the interactions between nicotine and stress during adolescence.

## Conclusion

This study provides insights into the behavioral and molecular consequences of chronic nicotine exposure in adolescent male mice exposed to social defeat stress. Our findings revealed that chronic social stress increased locomotor activity in aggressive mice while inducing anxiety and depression-like behaviors in both aggressive and defeated groups. Interestingly, nicotine exerted dose-dependent effects on the experimental groups studied. Low dose of nicotine (0.1 mg/kg) demonstrated anxiolytic and antidepressant like effects, particularly in defeated mice, whereas a higher dose (1 mg/kg) generally exacerbated depressive-like behavior, notably in control and defeated groups.

These behavioral changes were in parallel with the alterations reported in the expression of the Na+/K + ATPase α2 isoform in brain striatum. Elevated expression of the α2 isoform was linked to the anxiety and depressive like effects observed in defeated mice and defeated groups that received higher nicotine doses. In contrast, reduced expression in the defeated group receiving the lower nicotine dose aligned with the observed behavioral improvements, suggesting a potential role of this isoform in stress and nicotine interactions.

Altogether, our data highlight the importance of dose, duration, and behavioral parameters in understanding nicotine’s impact on emotional and cognitive processes under chronic stress. These findings also support the hypothesis that Na+/K + ATPase α2 may serve as a potential molecular mediator of stress-related disorders and could serve as a potential target for therapeutic intervention. Future research should aim to unravel the cellular mechanisms driving these interactions and investigate sex-specific and developmental variations to enhance knowledge relevance to human health.

## Data Availability

No datasets were generated or analysed during the current study.

## Abbreviations

SCM: Sensory Contact Model
i.p.: Intraperitoneal
OF: Open Field
EPM: Elevated Plus Maze
FST: Forced Swim Test
qRT-PCR: Quantitative Real-Time Polymerase Chain Reaction
SEM: Standard Error of the Mean
ANOVA: Analysis of Variance
CUMS: Chronic Unpredictable Mild Stress
mRNA: Messenger Ribonucleic Acid
Na⁺/K⁺ ATPase: Sodium-Potassium Adenosine Triphosphatase
qRT-PCR: Quantitative Reverse Transcription Polymerase Chain Reaction
